# The Reversible Electron Transfer Within Stimuli-Responsive Hydrochromic Supramolecular Material Containing Pyridinium Oxime and Hexacyanoferrate (II) Ions

**DOI:** 10.3390/molecules29235611

**Published:** 2024-11-27

**Authors:** Blaženka Foretić, Teodoro Klaser, Juraj Ovčar, Ivor Lončarić, Dijana Žilić, Ana Šantić, Zoran Štefanić, Alen Bjelopetrović, Jasminka Popović, Igor Picek

**Affiliations:** 1 Department of Chemistry and Biochemistry, School of Medicine, University of Zagreb, Šalata 3, HR-10000 Zagreb, Croatia; bforetic@mef.hr; 2 Physics Department, Faculty of Science, University of Zagreb, Bijenička cesta 32, HR-10000 Zagreb, Croatia; tklaser@phy.hr; 3 Ruđer Bošković Institute, Bijenička cesta 54, HR-10000 Zagreb, Croatia; juraj.ovcar@gmail.com (J.O.); ivor.loncaric@irb.hr (I.L.); dzilic@irb.hr (D.Ž.); ana.santic@irb.hr (A.Š.); zoran.stefanic@irb.hr (Z.Š.); alen.bjelopetrovic@irb.hr (A.B.)

**Keywords:** pyridinium oxime, hexacyanoferrate (II), charge-transfer complexes, charge separation, supramolecular complexes, electron transfer

## Abstract

The structural and electronic features of the stimuli-responsive supramolecular inter-ionic charge-transfer material containing electron accepting *N*-benzylyridinium-4-oxime cation (BPA4^+^) and electron donating hexacyanoferrate (II) are reported. The study of reversible stimuli-induced transformation between hydrated reddish-brown (BPA4)_4_[Fe(CN)_6_]·10H_2_O and anhydrous blue (BPA4)_4_[Fe(CN)_6_] revealed the origin of observed hydrochromic behavior. The comparison of the crystal structures of decahydrate and anhydrous phase showed that subsequent exclusion/inclusion of lattice water molecules induces structural relocation of one BPA4^+^ that alter the donor-to-acceptor charge-transfer states, resulting in chromotropism seen as reversible reddish-brown to blue color changes. The decreased donor-acceptor distance in (BPA4)_4_[Fe(CN)_6_] enhanced charge-transfer interaction allowing charge separation via one-electron transfer, as evidenced by in-situ ESR and FTIR spectroscopies. The reversibility of hydrochromic behavior was demonstrated by in-situ HT-XRPD, hot-stage microscopic and in situ diffuse-reflectance spectroscopic analyses. The insight into electronic structural features was obtained with density functional theory calculations, employed to elucidate electronic structure for both compounds. The electrical properties of the phases during dehydration process were investigated by temperature-dependent impedance spectroscopy.

## 1. Introduction

Organic and metal-organic supramolecular materials incorporating quaternary pyridinium salts (QPS) are continuously gathering immense attention due to modularity of their building blocks, inherent reversibility and overall versatility. The utility of supramolecular *N*-substituted pyridinium- and dipyridinium-based materials stems from possible various combinations of different noncovalent interactions from which they assemble, endowing them with great variety of dynamic and chromic properties that can be designed and controlled in both, solution and solid state [1,2]. The electron-deficient *N*-substituted pyridinium system possesses pronounced electron-accepting capability and a strong propensity to form stable, intensely colored radical species through facile and reversible one-electron transfer (ET) from an electron-donor [3,4,5], which enabled their application in electrochromic devices [6], catalysis [7,8] and molecular machines [9]. In addition, the distinctive chromic properties of a single pyridinium cation, manifested in observed red-shift of an absorption band after forming a radical species represent an efficient sensing strategy for quantifying pharmacologically active pyridinium oximes, namely pralidoxime [10]. Introducing non-covalent charge-transfer (CT) interactions between an organic electron-donor and pyridinium type electron-acceptor brings a multitude of advantages because of their stimuli-responsive thermochromic/hydrochromic [11,12,13], photochromic [14,15] and piezochromic [12,15,16] properties. These optical properties are further enhanced when external stimuli lead to structural perturbations that allow intramolecular charge-separation via one-electron transfer through delicate adjustment of HOMO-donor and LUMO-acceptor orbitals [15,17]. The utilization of highly charged electron-donating cyanoferrate platforms to build hybrid, metal-organic supramolecular charge-transfer complexes (CTCs) has attracted much attention, especially upon incorporating 4,4′-dipyridinium dication (viologen) and its derivatives [18,19,20,21]. Numerous viologen-based supramolecular hexacyanoferrate (II), [Fe(CN)_6_]^4−^, CTCs were synthesized in order to enhance photo- and thermally-induced charge separation through ET [22,23] and to additionally stabilize single monomeric radical species [24]. In addition, [Fe(CN)_6_]^4−^ and related [Fe(CN)_6–n_(HCN)_n_]^(4−n)−^ platforms, as a potential acceptors of six or more bifurcated H-bonds, are excellent building blocks in creating functional multidimensional H-bond networks [25,26,27].

Among diverse subclasses of QPSs, pyridinium oximes have been well known for decades as functional compounds, which display various modes of action. As powerful α-nucleophiles, they are well known esterolytic agents [28,29], pharmacologically recognized as potent reactivators of acetylcholinesterase (AChE) initially inhibited by organophosphorus poisons [30,31]. The non-AChE-reactivation effects of pyridinium oximes were also investigated with the focus on their electrochemical properties as another aspect of their antidotal bioactivity [32]. These studies confirm the intrinsic possibility of pyridinium oximes to form corresponding radical species in a one-electron reduction process. The σ-donor and π-acceptor potential of pyridinium oximes was established by equilibrium and kinetic studies of the aqueous substitution reactions with aquapentacyanoferrate(II), [Fe(CN)_5_(H_2_O)]^3−^, ion, as well as spectroscopic characterization of the isolated inner-sphere metal-to-ligand CTCs [33,34]. Focused on electron- and charge-transfer processes, mono- and bispyridinium-4-oximes are recently recognized as promising electron-accepting building blocks for the design of stimuli-responsive 2D- and 3D-supramolecular inter-ionic CTCs with the [Fe(CN)_6_]^4−^ ion as an electron donor [35,36]. In addition, the reversible hydrochromic behavior observed when bis-pyridinium-4-oxime Toxogonin^®^ was employed [36] makes them promising candidates to build-up new environment-responsive materials. In the case of CTC between *N*-benzylpyridinium-4-oxime (BPA4^+^) and [Fe(CN)_6_]^4−^ ion, the resolved crystal structure of the parent, hydrated phase, (BPA4)_4_[Fe(CN)_6_]·10H_2_O [35] emphasized lattice water molecules incorporated within hydrophilic pockets as essential structural feature for the formation of H-bonded 3D-network. In addition, the TG/DTA analysis showed stepwise loss of lattice water molecules over the temperature range 35–90 °C while the decomposition started above 140 °C. The color change observed upon partial extraction of weakly bound water molecules and the formation of intermediate hydrate (BPA4)_4_[Fe(CN)_6_]·2H_2_O motivated us to further investigate this transition between hydrated and anhydrous phases.

Here, we present the comprehensive structural and spectroscopic solid-state study, complemented with density functional theory calculations, of the stimuli-responsive CTC between BPA4^+^ and [Fe(CN)_6_]^4−^ ion. Structural formula of BPA4^+^ is given in Figure S1. The XRD structural analysis of anhydrous phase (BPA4)_4_[Fe(CN)_6_] and various spectroscopic techniques employed in our research provided insight into observed reversible hydrochromic behavior of isolated complex. The optical properties are result of distinct interplay between existing charge-transfer and dehydration-induced electron-transfer, which led to the formation of BPA4 radical and hexacyanoferrate( III) species within a material.

## 2. Results and Discussion

**Figure 1:**
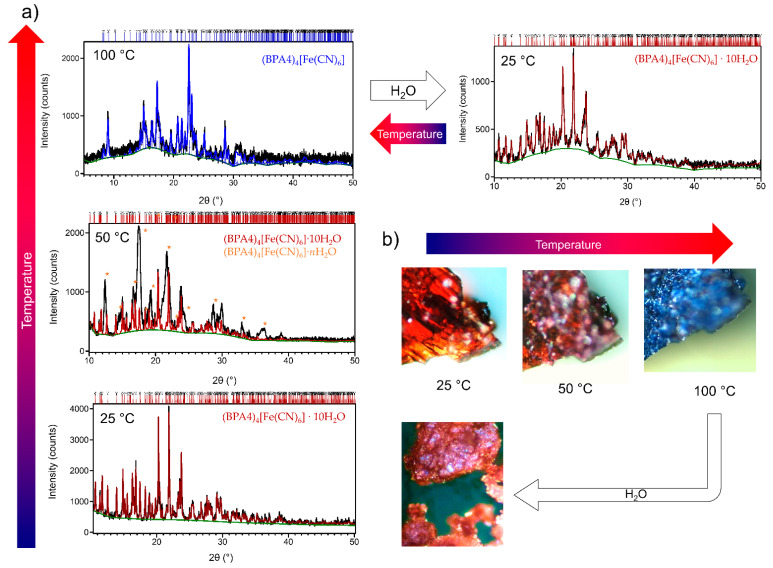
(**a**) Rietveld refinement on in-situ HT-XRPD data collected at 25, 50 and 100 °C. Experimental diffraction pattern is shown as black line. Calculated patterns for (BPA4)_4_[Fe(CN)_6_]·10H_2_O and (BPA4)_4_[Fe(CN)_6_] are represented by red and blue lines, respectively. Calculated background is given by green line. Diffraction lines corresponding to (BPA4)_4_[Fe(CN)_6_]·*n*H_2_O are marked with orange asterisk. (**b**) Hot-stage microscopy pictures of reversible hydrochromic behavior.

The in-situ high-temperature X-ray powder diffraction (in-situ HT-XRPD) patterns and hot-stage microscopy images of as-prepared (BPA4)_4_[Fe(CN)_6_]·10H_2_O collected at 25, 50 and 100 °C are shown in Figure 1.

Results of the Rietveld refinements that were carried out on collected in-situ HT-XRPD data provided an insight into the thermally-induced structural evolution of as-prepared (BPA4)_4_[Fe(CN)_6_]·10H_2_O sample. Pattern collected at room temperature (25 °C) shows only the diffraction lines that correspond to (BPA4)_4_[Fe(CN)_6_]·10H_2_O and no additional crystalline phases were observed. Rietveld refinement at 25 °C was performed by using the structure of (BPA4)_4_[Fe(CN)_6_]·10H_2_O as previously reported [35]. As temperature increases crystals are slowly starting to lose water molecules; at 50 °C, the diffraction lines of decahydrate are still present however the appearance of additional diffraction lines, most probably corresponding to intermediate hydrate, were also observed (denoted by *). At 100 °C, only the diffraction lines corresponding to anhydrous (BPA4)_4_[Fe(CN)_6_] are present. Rietveld refinement at 100 °C was performed by using the structure of as determined from single crystal diffraction during this study. The structure of anhydrous (BPA4)_4_[Fe(CN)_6_] is shown in Figure 4b while the corresponding crystal data are given in Table S1. Asymmetric unit of (BPA4)_4_[Fe(CN)_6_] contain of one half of [Fe(CN)_6_] anion and two BPA4 cations. There are two symmetry independent hydrogen bonds, both between Fe(CN)_6_ and BPA4 species: O1A···N1 (2.66 Å) and O1B···N3 (2.70 Å). The crystal packing is then realized over [Fe(CN)_6_] units, where Fe atoms lie on the centers of symmetry in the P-1 space group as shown in Figure S2. The results of Hirshfeld surface analysis for both, (BPA4)_4_[Fe(CN)_6_]·10H_2_O and (BPA4)_4_[Fe(CN)_6_], that provide additional insight into intermolecular interactions of hexacyanoferrate (II) anion, are given in Figure S3. From hot-stage microscopy (Figure 1b) we can observe the change of color, from reddish-brown to blue, that accompanies the loss of water molecules. This hydrochromic phenomenon is reversible; upon adding the drop of water to the blue crystals of anhydrous phase, it swiftly transforms back to reddish-brown crystals of decahydrate phase.

**Figure 2:**
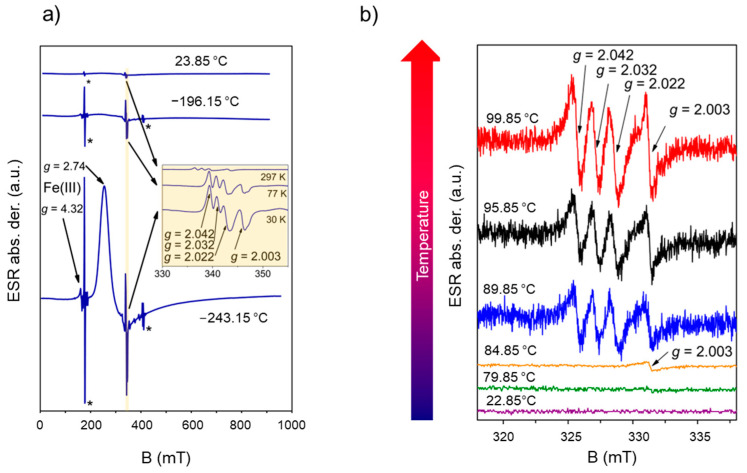
(**a**) ESR spectra of anhydrous (BPA4)_4_[Fe(CN)_6_] recorded at selected temperatures (microwave frequency ~9.7 GHz). Inset shows enlarged parts of spectra in the narrow magnetic field range 330–355 mT. Lines marked by asterisks belong to the ESR cavity. (**b**) Temperature dependence of ESR spectra of the as-prepared sample (BPA4)_4_[Fe(CN)_6_]·10H_2_O from room temperature up to 100 °C (microwave frequency ~9.3 GHz).

Both complexes were investigated by means of electron spin resonance (ESR) spectroscopy. The reddish-brown complex (BPA4)_4_[Fe(CN)_6_]·10H_2_O was ESR silent from room temperature down to liquid helium temperature, as expected for non-Kramer Fe^2+^ compounds in X-band ESR spectroscopy [37,38]. Contrary, the spectra of (BPA4)_4_[Fe(CN)_6_], shown in Figure 2a, provided evidence of paramagnetic species present within the solid sample. The strongest signal that appeared below −193.15 °C at *g* = 2.74 is assigned to Fe^3+^ center in low-spin state *S* = 1/2 as a clear evidence of the existence of [Fe^III^(CN)_6_]^3−^ ion [22,39,40]. However, high-field signal at *g* = 0.83 observed in the spectrum of viologen-based hexacyanoferrate CTC [22] was not detected in applied magnetic field range (5–950 mT). The second signal consists of three lines marked with *g* = 2.042, 2.032, 2.022, and it could be detected in the whole temperature range. This signal is related to the unpaired electron in presence of nitrogen nucleus with nuclear spin *I* = 1 [41]. Also, observed weak signal detected below −193.15 °C at *g* = 4.32 assigned to high spin Fe^3+^ ions [42] and signal at *g* = 2.003 is associated with free radical. Both signals likely originate from the grain boundaries.

The evolution of ESR signals upon thermally-induced dehydration of (BPA4)_4_[Fe(CN)_6_]·10H_2_O was monitored by in-situ ESR spectroscopy, performed on as-prepared sample in the temperature range of ~23–100 °C (Figure 2b). The sample remained ESR silent up to ~80 °C while at ~85 °C, the signal of free BPA4 radical with *g* = 2.003 appeared. Above ~90 °C, the triplet with *g* = 2.042, 2.032 and 2.022 could be clearly detected. As expected, the signals of Fe^3+^ ions due to fast relaxation were not detected at high temperatures. The ESR study clearly revealed that thermal stepwise extraction of lattice water molecules promotes the generation of BPA4^●^ radical and [Fe^III^(CN)_6_]^3−^ species through one-electron transfer from [Fe^II^(CN)_6_]^4−^ to BPA4^+^.

**Figure 3:**
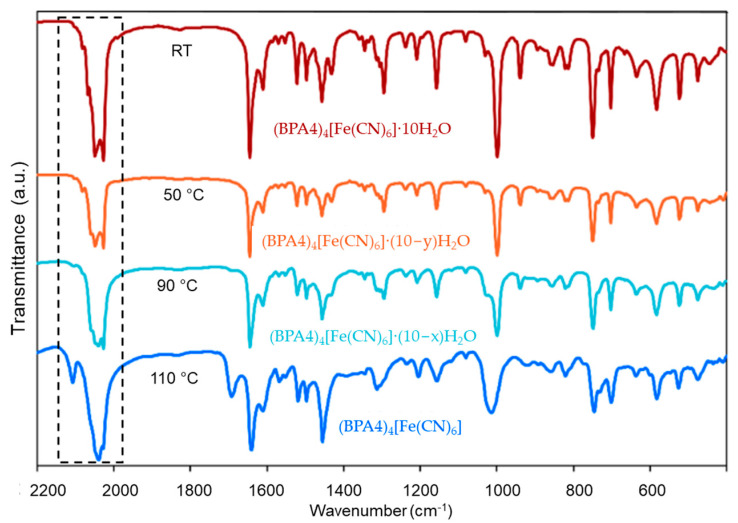
FTIR spectra recorded at RT of as-prepared (BPA4)_4_[Fe(CN)_6_]·10H_2_O (reddish-brown), first intermediate hydrate (orange), second intermediate hydrate (light blue) and anhydrous (BPA4)_4_[Fe(CN)_6_] (blue).

Further insight into dehydration-promoted electron-transfer was provided by the comparative FTIR study of the changes observed in the FTIR spectrum of the as-prepared (BPA4)_4_[Fe(CN)_6_]·10H_2_O upon temperature-induced dehydration. FTIR spectra, shown in Figure 3, recorded at ambient temperature after heating the parent complex to 50 °C, 90 °C and 110 °C, revealed several less hydrated phases intermediate and the final anhydrous (BPA4)_4_[Fe(CN)_6_].

The observed vibrational spectra showed strong ν(C≡N) bands in the 2020–2080 cm^−1^ region characteristic for [Fe^II^(CN)_6_]^4−^ ion as was found for other pyridinium oxime- [35,36] and bipyridinium-based hexacyanoferates (II) [18,19,22]. Furthermore, the oxime ν(C=N) band observed at 1643 cm^−1^ with significant ν(C=N) and ν(C=C) pyridinium ring contributions [43] is characteristic for a non-radical *N*-substituted pyridinium oximes [34]. The vibrational spectrum of anhydrous phase evidenced that even within the vibrational time-scale the apparent hydrochromic behavior involves dehydration-induced electron-transfer causing charge separation and formation of [Fe^III^(CN)_6_]^3−^ and BPA4^●^ radical. Namely, a new, well-resolved ν(C≡N) band at 2108 cm^−1^ is consistent with the presence of [Fe^III^(CN)_6_]^3−^ ion within (BPA4)_4_[Fe(CN)_6_] [44,45]. The domination of the ν(C≡N) band at 2040 cm^−1^ over the ν(C≡N) band at 2108 cm^−1^ is in accordance with small amount of [Fe^III^(CN)_6_]^3−^ present within the anhydrous phase, with respect to [Fe^II^(CN)_6_]^4−^. Plausible oxidation of [Fe^II^(CN)_6_]^4−^ to [Fe^III^(CN)_6_]^3−^ by molecular oxygen is discarded since the thermal analysis of K_4_[Fe^II^(CN)_6_]·3H_2_O showed that oxidation of Fe^2+^ to Fe^3+^ takes place above 350 °C [46]. The fact that evolution of ESR signals from [Fe^III^(CN)_6_]^3−^ and BPA4^●^ radical, detected above 90 °C corresponds very well with the first appearance of FTIR bands at 2108 cm^−1^ and 1690 cm^−1^ strongly suggests that the band at 1690 cm^−1^ corresponds to ν(C=N^●^) and ν(C=C) stretching vibrations within BPA4^●^ radical.

**Figure 4:**
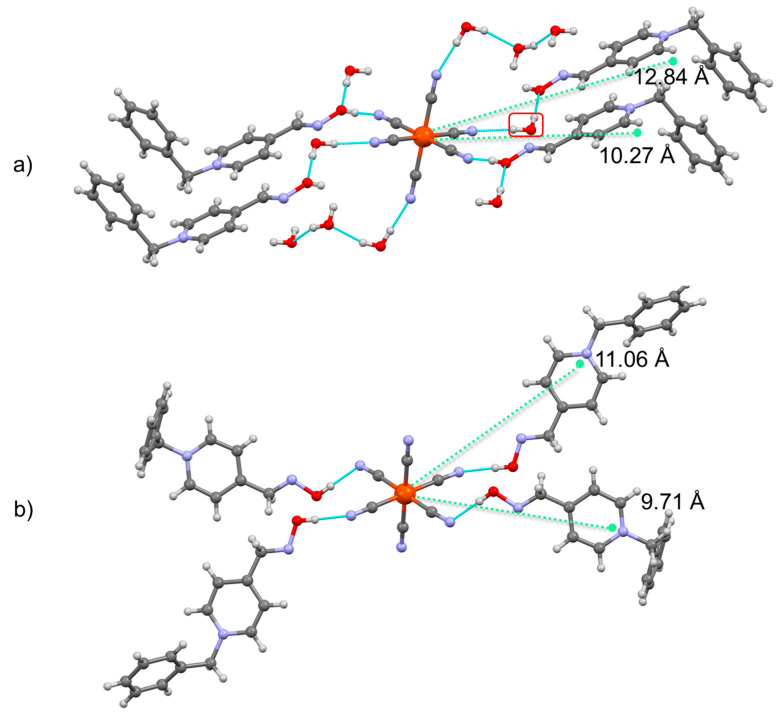
Structures of (**a**) (BPA4)_4_[Fe(CN)_6_]·10H_2_O and (**b**) (BPA4)_4_[Fe(CN)_6_]. Iron atoms are shown as orange balls while carbon, oxygen, nitrogen and hydrogen are shown as grey, red, blue and white balls, respectively. Hydrogen bonds are given by dashed turquoise lines. Distances between centroids are given by green dashed lines.

Changes in electronic characteristics found by ESR and complemented with FTIR upon dehydration are reflection of the structural modification evidenced by inspection of crystalline phases involved in the reversible hydrochromic transition (Figure 4).

In decahydrate phase, one BPA4^+^ cation is connected to the CN^−^ ligand via hydrogen bonding and this type of bonding mode remains similar in the case of anhydrous structure. From the calculated distances between centroids, it can be observed that this BPA4^+^ cation within anhydrous phase is closer to Fe-core (9.711 Å compared to 10.267 Å for decahydrate phase). However, significant difference is noted for the second BPA4^+^ cation; in the case of decahydrate, second BPA4^+^ cation is connected by hydrogen bonding to water molecule while in the anhydrous phase, as expected, hydroxyl group of oxime moiety establishes a direct hydrogen bond with CN^−^ ligand. While the transition from decahydrate phase to anhydrous phase includes the loss of all water molecules, it is quite plausible that, in fact, removal of this particular water molecule plays a pivotal role for the pronounced charge separation within this supramolecular assembly in the case of anhydrous phase. From electron-donor and electron-acceptor distance, defined as a distance between two centroids (centroid 1 is calculated for 13 atoms of hexacyanoferrate core and centroid 2 is calculated for 15 atoms of BPA4^+^) we can observe that distance between centroids in anhydrous structure decreased significantly upon the loss of water molecule (11.056 Å compared to 12.837 Å). From the in-situ ESR experiment, complemented with FTIR study we demonstrated that, upon dehydration, only the loss of water molecules above 90 °C leads to electron-transfer. Thus, the formation of direct hydrogen bond between CN^−^ ligand and the second BPA4^+^ by extraction of bridging water molecule causes delicate structural relocation of the donor that enables the electron- transfer.

**Figure 5:**
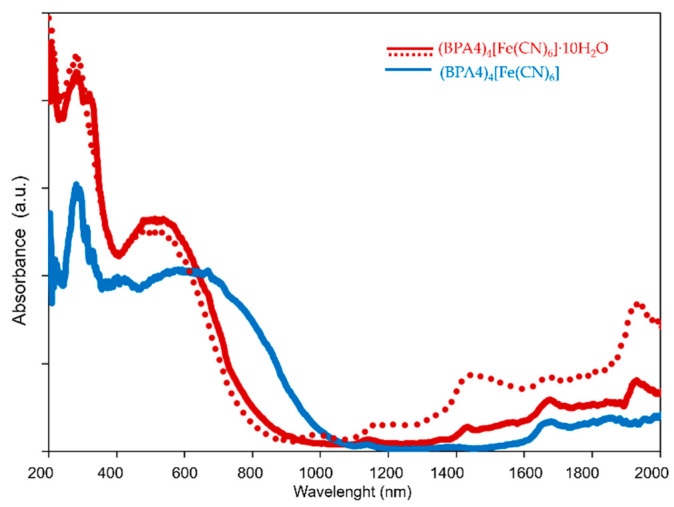
UV/Vis/NIR spectra of as-prepared (BPA4)_4_[Fe(CN)_6_]·10H_2_O (reddish-brown curve), thermally dehydrated (BPA4)_4_[Fe(CN)_6_] (blue curve) and rehydrated (BPA4)_4_[Fe(CN)_6_]·10H_2_O (dotted reddish-brown curve) recorded at room temperature.

Since, the dehydration-induced transition from reddish-brown (BPA4)_4_[Fe(CN)_6_]·10H_2_O to blue (BPA4)_4_[Fe(CN)_6_] is followed by a significant change in electronic structures the reversibility of this process is supported by the comparison of their Uv/Vis/NIR electronic absorption spectra obtained for one dehydration/rehydration cycle by in-situ diffuse-reflectance spectroscopy (Figure 5).

**Figure 6:**
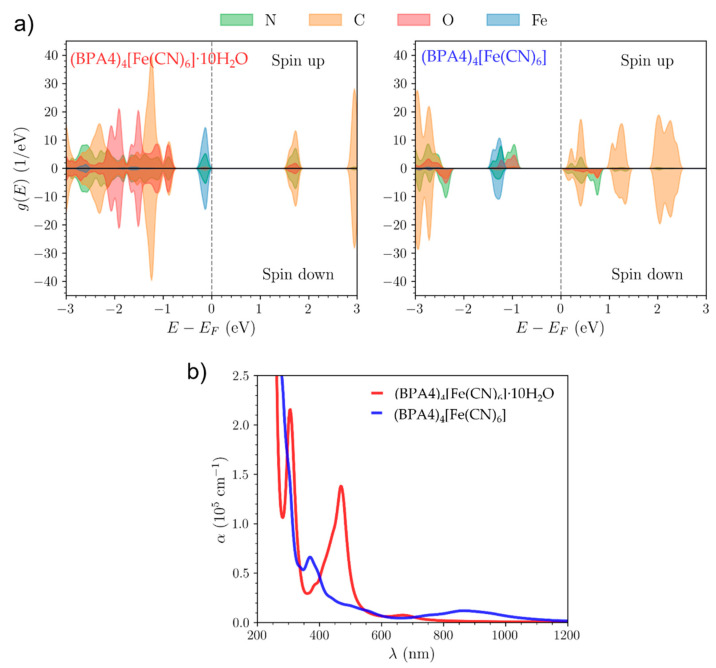
(**a**) Electronic density of states projected on atomic orbitals for (BPA4)_4_[Fe(CN)_6_] ∙10H_2_O (left panel) and (BPA4)_4_[Fe(CN)_6_] (right panel); (**b**) The absorption spectra of (BPA4)_4_[Fe(CN)_6_] ·10H_2_O and (BPA4)_4_[Fe(CN)_6_] as calculated using DFT.

The absorption spectrum of as-prepared (BPA4)_4_[Fe(CN)_6_]·10H_2_O, discussed in our previous work [35], is dominated by the strong and well-resolved inter-ionic charge-transfer (IICT) band in the Vis region with maximum centered at 563 nm, originating from d(t_2g_^6^)p(π^0^)*→d(t_2g_^5^)p(π^1^)* transition. The complete temperature-induced dehydration and formation of (BPA4)_4_[Fe(CN)_6_] results in rather complicated electronic absorption fingerprint within Vis/NIR spectral region. Besides unchanged UV-absorption bands originated from electronic transitions within BPA4^+^ and [Fe^II^ (CN)_6_]^4−^ constituents, a new, band at 405 nm is observed, while the IICT band of anhydrous phase is broadened and red-shifted with estimated maximum at 670 nm. Such spectral changes are in accordance with detected electron-transfer, giving raise to new additional set of electronic transitions occurring within BPA4^●^ and [Fe^III^(CN)_6_]^3−^ species. The dehydration-induced red shift of the IICT band, but without change in its width is also observed in a closely related and redox-inert bis-pyridinium oxime-based hexacyanoferrate (II) [36]. Thus, the red shift of the IICT band of anhydrous phase with respect to decahydrate is a consequence of enhanced donor-acceptor CT interaction due to substantial decrease in BPA4^+^····[Fe(CN)_6_]^4−^ distance while the band broadening reflects the presence of highly absorbing radical BPA4^●^. This is confirmed with DFT calculations, which show the reduction in the band gap upon dehydration, and that lowest energy transitions originate solely from BPA4^●^ radical species (Figure 6). Moreover, the fact that isolated bipyridinium mono radical species show several absorption CT bands in Vis/NIR spectral region in both, solution and solid state [47,48] further accounts for the observed broadening of the IICT band. In line with these observations, the absorption band at 405 nm, otherwise absent in spectra of ESR-silent pyridinium oxime hexacyanoferrate (II) complexes [35,36] incorporates contributions from electron transitions within BPA4^●^ and [Fe^III^(CN)_6_]^3−^, the latter being characterized by the several absorption bands in 250–450 nm region, assigned to d−d and metal-to-ligand CT transitions [49]. Furthermore, one dehydration/rehydration cycle, monitored by the disappearance and reappearance of specific NIR bands of water at 1430 nm (first overtone ν(OH) and 1930 nm (ν(OH)+*δ*(OH)), ends in complete restoration of (BPA4)_4_[Fe(CN)_6_]·10H_2_O spectrum. This conforms to HT-XRPD results and confirms a reversible solid-state hydrochromic behavior of this material and the ability to swiftly switch from redox-inert to redox-active phase.

To supplement this experimental results and to provide further insight into the origin of observed absorption bands for (BPA4)_4_[Fe(CN)_6_], we performed comparative density functional theory (DFT) calculations of the density of states using the crystallographic data of both, (BPA4)_4_[Fe(CN)_6_]·10H_2_O and (BPA4)_4_[Fe(CN)_6_].

The difference in the CT nature of (BPA4)_4_[Fe(CN)_6_]·10H_2_O versus (BPA4)_4_[Fe(CN)_6_] may be clearly seen from the calculated ground state electronic density (Figure S4). In (BPA4)_4_[Fe(CN)_6_], all CN^−^ ligands are hydrogen-bonded to the BPA4^+^ cations, while in (BPA4)_4_[Fe(CN)_6_]·10H_2_O, we observe no significant charge transfer between the ligands and the water molecules. Electronic density of states projected on atomic orbitals and the absorption spectra are presented on Figure 6.

Projections to atomic orbitals show that bands shown in Figure 6a are formed of *p*-orbitals of C, N and O and Fe *d*-orbitals. Electronic structure of (BPA4)_4_[Fe(CN)_6_]·10H_2_O is characterized by the valence band localized on [Fe(CN)_6_] and a conduction band localized on BPA4 that is hydrogen-bonded to the to the CN^−^ ligand. Bands below the valence band have contributions from all BPA4 molecules. All electrons are paired, i.e., there is no spin polarization in agreement with ESR results.

(BPA4)_4_[Fe(CN)_6_] is, on the other hand, spin-polarized, with both the top of the valence band and bottom of the conduction band found on the BPA4. The spin polarization comes from lifting the degeneracy of the hybridized *p*-orbitals localized on the N and O atoms of BPA4 molecules within a single BPA4 molecule, again in agreement with ESR results. Due to the equilibrium nature of calculations, Fe^3+^ ions are not obtained.

The calculated absorption spectra of (BPA4)_4_[Fe(CN)_6_]·10H_2_O and (BPA4)_4_[Fe(CN)_6_] are shown on Figure 6b. Note that this is a crude single particle estimate of optical spectra with the well-known systematic underestimation of band gaps in semilocal DFT. Lowest energy optical peak for (BPA4)_4_[Fe(CN)_6_]·10H_2_O is estimated at around 670 nm stemming from the transitions from the valence band localized on the [Fe(CN)_6_] to the conduction band localized on BPA4 that is hydrogen bonded to the CN^−^ ligand. For (BPA4)_4_[Fe(CN)_6_], consistent with the reduction in the band gap, the lowest energy transitions result in a broad peak at 870 nm. These peaks can be correlated to the experimentally observed absorption bands that are responsible for the change in color.

**Figure 7:**
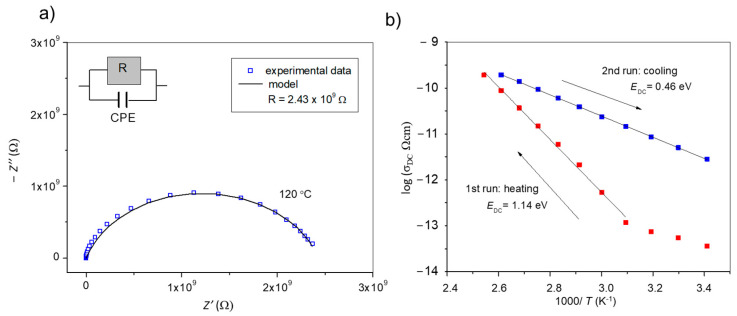
(**a**) Complex impedance plot and corresponding equivalent circuit for anhydrous phase (BPA4)_4_[Fe(CN)_6_] at 120 °C. (**b**) DC conductivity as a function of reciprocal temperature during dehydration of (BPA4)_4_[Fe(CN)_6_]·10H_2_O.

Above results are supplemented by the electrochemical impedance spectroscopy. A typical experimental impedance of anhydrous phase (spectrum measured at 120 °C) exhibits a semicircle in the complex plot as shown in Figure 7a. Such impedance data can be well approximated by the equivalent electrical circuit consisting of a parallel combination of resistor (R) and constant phase element (CPE). The value of the electrical resistance obtained by the complex nonlinear least-squares fitting of the impedance spectrum at 120 °C is shown in the legend. Similar shape of the complex impedance plots was obtained for each measured temperature, so similar fitting was performed to determine the electrical resistance of the compound during phase transition.

Figure 7b shows an Arrhenius plot of DC conductivity of (BPA4)_4_[Fe(CN)_6_]·10H_2_O during phase transition to anhydrous form. The DC conductivity of (BPA4)_4_[Fe(CN)_6_]·10H_2_O at the beginning of measurement at 20 °C is very low (σ_DC_ = 3.61∙10^−14^ (Ω cm)^−1^) and shows a weak temperature dependence up to 50 °C. However, in heating from 50 °C to 120 °C, DC conductivity strongly increases exhibiting Arrhenius temperature dependence with the activation energy of 1.14 eV. The observed strong increase of DC conductivity in this temperature range is related to the structural changes in the sample, i.e., loss of water molecules and formation of intermediate hydrate as detected by HT-XRPD. Upon heating the intermediate hydrate completely transforms to a significantly more conducting anhydrous phase, which can be further followed in subsequent cooling run. Finally, it can be observed that the DC conductivity of the (BPA4)_4_[Fe(CN)_6_] as mixed valence [Fe^II^(CN)_6_]^4−^/[Fe^III^(CN)_6_]^3−^ compound is at 20 °C two orders of magnitude higher (σ_DC_ = 3.22·10^−12^(Ωcm)^−1^) than that of the starting single valence (BPA4)_4_[Fe(CN)_6_]·10H_2_O. The high conductivity of anhydrous compound is a direct consequence of the presence of [Fe^II^(CN)_6_]^4−^ and [Fe^III^(CN)_6_]^3−^ ions which enables the transfer of electrons between them. Also, the facilitated electronic transport in the anhydrous phase is reflected in drastically lower activation energy, E_DC_ = 0.46 eV. Here, it is interesting to note that the loss of crystallization water, which triggers the oxidation of Fe^2+^ ions in (BPA4)_4_[Fe(CN)_6_]·10H_2_O, and hence indirectly facilitates the electrical conductivity due to electronic transport between iron ions, did not resulted in a decrease in the DC conductivity during heating, demonstrating that the proton transfer in this compound is negligible. Also, it should be noted that the reversibility of the decahydrate to anhydrous transformation could not have been observed in these measurements since the heating-cooling cycle was performed in the atmosphere of dry nitrogen.

## 3. Materials and Methods

### 3.1. Experimental Techniques

Single crystal X-ray diffraction (XRD) data were collected from suitable single crystals on an Oxford Diffraction Xcalibur single-crystal diffractometer (Rigaku Oxford Diffraction, Abingdon, United Kingdom) with Ruby CCD detector and using Cu Kα radiation. The software used fo data collection and reduction was CrysAlisPro version 1.171.39.46 (Rigaku OD, 2018) [50]. The crystal was kept at 293(2) K during data collection. Using Olex2 [51], the structure was solved with the SHELXT [52] structure solution program using Intrinsic Phasing and refined with the SHELXL [53] refinement package using Least Squares minimisation. All non-hydrogen atoms were refined anisotropically. Hydrogen atoms bound to carbon atoms were placed in calculated positions. The crystallographic data are summarized in Table S1. The crystal structure is deposited in the Cambridge Structure Database under the deposition number CCDC Deposition Number 2384887.

Temperature-induced structural/compositional changes of (BPA4)_4_[Fe(CN)_6_] were investigated by the means of the in-situ high temperature X-ray powder diffraction (in-situ HT-XRPD) using the Philips MPD 1880 counter diffractometer (Philips, Eindhoven, Netherlands) with CuK_α_ radiation equipped with Anton Paar high temperature chamber. Diffraction data were collected in the 2*θ* range 10–50° with the step 0.02 ° and fixed counting time of 5 s per step. Structure refinement was carried out by the Rietveld method with Pseudo-Voigt function used to describe the diffraction line profiles while the polynomial model was used for defining the background. All atoms were assumed to show isotropic vibration modes. All profile parameters: zero shift, scale factor, half-width parameters (U, V, W), asymmetry parameters and peak shape parameters were simultaneously refined during the Rietveld regiment together with unit-cell parameters. Contrary to this standard refinement procedure that was applied for data collected at 25 and 100 °C, during the Rietveld refinement on data collected at 50 °C some constraints have been introduced, namely, parameter W was constrained to the final value obtained during the refinement of data collected at 25 °C, while U and V were not refined. Since the structure of this intermediate phase is unknown quantitative analysis of data collected at 50 °C was not possible. Structure of (BPA4)_4_[Fe(CN)_6_]·10H_2_O was used for structural refinement of data at 25 and 50 °C while the structure of anhydrous phase (BPA4)_4_[Fe(CN)_6_] was used as a structural model for refinement of data at 100 °C.

The program Crystal Explorer 21.5 [54] was used to perform Hirshfeld surface analysis to gain additional insight into intermolecular interactions of hexacyanoferrate (II) ion. The calculations were performed using cif-files of the prepared materials. The normalized contact distance *d*_norm_ which is related to *d*_e_ (distance from the point of the nearest nucleus external to the surface) and *d*_i_ (distance to the nearest nucleus internal to the surface), is mapped onto the Hirshfeld surface using a red-blue-white color scheme: red regions correspond to shorter contacts with negative *d*_norm_ value, the blue areas correspond to longer contacts with positive *d*_norm_ value, and the white regions correspond to those where the distance of the contacts is precisely the Van der Waal’s separation (*d*_norm_ value of 0). The curvedness is a measure of the shape of the surface area of the molecule. The *d*_norm_ surfaces were mapped over the range of −0.781·4 a.u. to 1.0381 a.u. for (BPA4)_4_[Fe(CN)_6_]·10H_2_O and from −0.7073 a.u. to 1.1983 a.u. for (BPA4)_4_[Fe(CN)_6_]. The curvedness was mapped over the range of −4.000 a.u. to 4.000 a.u. for both structures.

Reversible hydrochromic dehydration/rehydration process was examined and recorded using Nikon Eclipse LV150NL optical microscope (Nikon corp. Nishioi, Shinagawa-ku, Tokyo, Japan) equipped with a Linkam THMS600 hot-stage and OPTOCAM-II color camera with a resolution of 1600 × 1200 pixels.

Electron spin resonance (ESR) spectroscopic study was performed on powder samples using an X-band Bruker Elexsys 580 FT/CW spectrometer (Bruker, Ettlingen, Germany), equipped with a standard Oxford Instruments model DTC2 temperature controller. The measurements were obtained at the microwave frequency around 9.7 GHz with the magnetic field modulation amplitude of 0.5 mT at 100 kHz. The spectra were recorded from room down to liquid helium temperature (297-5 K). ESR spectra above room temperature were recorded by a Varian E-9 spectrometer with the following parameters: microwave frequency around 9.3 GHz and magnetic field modulation amplitude of 1 mT at 100 kHz. The Varian spectrometer was equipped with a Bruker variable temperature unit ER 4111 VT. Liquid nitrogen was used for heating samples in the temperature range 25–100 °C.

FTIR spectra were recorded at room temperature in the range 2200–400 cm^−1^ using a PerkinElmer Spectrum Two FTIR spectrometer (PerkinElmer, Waltham, MA, USA) equipped with a diamond UATR accessory and a pressure arm with a force indicator. The sample preparation for monitoring spectral changes during the course of dehydration was performed as follows: the as-prepared (BPA4)_4_[Fe(CN)_6_]·10H_2_O sample was heated, each time up to different temperature (50, 90 and 110 °C) to obtain two intermediate hydrates and the anhydrous phase.

Diffuse reflectance spectra were recorded at room temperature in the range 200–2000 nm in the absorbance mode using a Shimadzu UV-3600 spectrophotometer (Shimadzu Corp., Kyoto, Japan) equipped with an integrated sphere and calibrated against a surface of barium sulfate for 100% reflectance. The in-situ experiment was performed as follows: the barium sulfate was placed in the holder and the as-prepared reddish-brown (BPA4)_4_[Fe(CN)_6_]·10H_2_O sample, previously ground to fine powder, was carefully adsorbed to the central position of the barium sulfate surface. After the spectrum is recorded, the as-prepared sample was heated to 110 °C and the spectrum of formed blue (BPA4)_4_[Fe(CN)_6_] is obtained. Then, one drop of water was carefully added to the blue anhydrous phase, left at room temperature for one hour and the spectrum of restored reddish-blue sample was recorded.

The electrical properties of compound (BPA4)_4_[Fe(CN)_6_]·10H_2_O during temperature-induced dehydration were measured by impedance spectroscopy using Novocontrol Alpha-N dielectric analyser (Novocontrol Technologies GmbH & Co. KG, Montabaur, Germany) in the frequency range from 0.01 Hz to 1 MHz and temperature range from 20 °C to 120 °C. For the measurements, the polycrystalline (BPA4)_4_[Fe(CN)_6_]·10H_2_O was pressed into a pellet and placed between brass electrodes. The impedance spectra were measured isothermally, first at temperatures in heating from 20 °C to 120 °C and then in cooling back to 20 °C. The temperature step in both, heating and cooling runs, was 10 °C. The impedance spectra were analysed by equivalent circuit modelling using the complex nonlinear least-squares fitting procedure (ZView software Ver 1.). From the value of the circuit parameter resistance (R) and electrode dimension (*A* is the electrode area and *d* is the sample thickness), the DC conductivity, *σ*_DC_, for each temperature was calculated according to relation: *σ*_DC_ = *d*/(*A*·*R*).

Elemental analysis was performed using a Perkin Elmer 2400 Series II CHNS elemental analyzer (PerkinElmer, Waltham, MA, USA) with an accuracy of ±0.3%.

### 3.2. Computational Details

All calculations were performed within the spin-polarized density functional theory (DFT) using the QuantumATK atomic-scale modeling software [55]. We used the Perdew-Burke-Ernzerhof (PBE) exchange-correlation functional [56] and norm-conserving pseudopotentials [57] with the local potential cutoff radius of 6.0 Bohr. A Hubbard-U correction of 3 eV was used for the *d* orbital of Fe. Integration in the Brillouin zone was performed using a Monkhorst-Pack uniform k-point grid [Monkhorst1976] with a density of 6 Å in each of the directions of the reciprocal cell. All atomic positions were relaxed with the convergence criterion of 0.05 eV/Å for the atomic forces. The absorption coefficient was calculated within linear response theory. We used a 5 × 5 × 5 Monkhorst-Pack k-point grid and included 50 bands below and 50 bands above the Fermi level in the Kubo-Greenwood formula for the susceptibility tensor, with the energy broadening parameter set to 0.1 eV.

### 3.3. Isolation of (BPA4)_4_[Fe(CN)_6_]

The as-prepared (BPA4)_4_[Fe(CN)_6_]·10H_2_O transformed to anhydrous phase (BPA4)_4_[Fe(CN)_6_] by extrication of water molecules induced by heating the parent precipitate up to 120 °C. The isolation procedure, structural, thermal and spectroscopic analyses of the parent (BPA4)_4_[Fe(CN)_6_]·10H_2_O were reported in our previous work [35]. The purity of (BPA4)_4_[Fe(CN)_6_] was confirmed by elemental and thermal analyses. Elemental analysis calculated (%) for C_58_H_52_N_14_O_4_Fe (*M*_r_ = 1064.99): C 65.41, H 4.92, N 18.41; found: C 65.24, H 4.87, N 18.33. Thermogravimetric analysis in the temperature range 25–120 °C: no significant weight loss was observed. FTIR (UATR, cm^−1^): *ν*(CN)_cyano_, 2039 (vs); *ν*(CN)_cyano_, 2107 (m); *ν*(C=N)_oxime_, 1641 (s); *ν*(CC, CN)_pyridinium ring_, 1610 (m), 1566 (w), 1519 (m); *ν*(N–O)_oxime_, 1012 (s); *δ*(Fe–C), 582 (m) cm^−1^.

## 4. Conclusions

In summary, a reversible solid-state hydrochromism of supramolecular donor-acceptor *N*-benzylpyridinium-4-oxime hexacyanoferrate charge-transfer complex has been investigated. The parent, redox-inert, decahydrate phase was transformed to redox-active anhydrous phase by thermally induced dehydration followed by a characteristic color change from reddish-brown to blue. Dehydration process caused enhancement of charge-transfer interaction allowing electron-transfer from hexacynoferrate (II) to pyridinium oxime and the formation of small amounts of BPA4^●^ radical and hexacynoferrate (III) within anhydrous phase. The electron-transfer, giving rise to color change, can be switched off by addition of water. This study presents the first example of stimuli-responsive redox-active metal-organic charge-transfer complex involving pyridinium oxime as an electron acceptor. The ease of its isolation and ability to switch on/switch off electron-transfer encourages us to continue to explore such pyridinium oxime-based materials providing a contribution to development of optical switches.

## Data Availability

Data are contained within the article and Supplementary Materials.

## Supplementary Materials

The following supporting information can be downloaded at: https://www.mdpi.com/article/10.3390/molecules29235611/s1, Figure S1: Structural formula of *N*-benzylpyridinium-4-oxime cation (BPA4^+^); Figure S2: Crystal packing of (BPA4)_4_[Fe(CN)_6_]; Table S1: Crystal data and structure refinement for (BPA4)_4_[Fe(CN)_6_]; Table S1: Crystal data and structure refinement for (BPA4)_4_[Fe(CN)_6_]; Figure S3: a) 2D Fingerprints plots and Hirshfeld surfaces of hexacyanoferrate ion mapped for b) *d*_norm_, and c) curvature of hexacyanoferrate(II) ion in crystal structures of up: (BPA4)_4_[Fe(CN)_6_]·10H_2_O and down: (BPA4)_4_[Fe(CN)_6_]; Figure S4: Isosurface of value 0.3 of the ground state electronic density for a) (BPA4)_4_[Fe(CN)_6_]·10H_2_O and b) (BPA4)_4_[Fe(CN)_6_]. In (BPA4)_4_[Fe(CN)_6_]·10H_2_O, no charge-transfer is observed between the CN- ligands and the water molecules (red dashed circle), while hydrogen bonds form between all ligands and the BPA4 cations (green dashed circles). For visual clarity, only a portion of the isosurfaces and the atoms in the unit cell is shown.
