# Supplementary material for: The Reversible Electron Transfer Within Stimuli-Responsive Hydrochromic Supramolecular Material Containing Pyridinium Oxime and Hexacyanoferrate (II) Ions
Source: Molecules. 2024 Nov 27;29(23):5611. doi: 10.3390/molecules29235611 (PMC11643671; doi:10.3390/molecules29235611)
Supplement: Supplementary file 1 [file molecules-29-05611-s001.zip › molecules-3318389-supplementary.pdf]

The Reversible Electron Transfer within Stimuli-responsive Hydrochromic Supramolecular Material containing Pyridinium Oxime and Hexacyanoferrate(II) Ions

Blaženka Foretić [1], Teodoro Klaser [2], Juraj Ovčar [3], Ivor Lončarić [3], Dijana Žilić [3], Ana Šantić [3], Zoran Štefanić [3], Alen Bjelopetrović [3], Jasminka Popović [3,*] and Igor Picek [1,*]

[1] Department of Chemistry and Biochemistry, School of Medicine, University of Zagreb, Šalata 3, HR-10000 Zagreb, Croatia; ipicek@mef.hr (I.P.); bforetic@mef.hr (B.F.)

[2] Physics Department, Faculty of Science, University of Zagreb, Bijenička cesta 32, HR-10000 Zagreb, Croatia; tklaser@phy.hr (T.K)

[3] Ruđer Bošković Institute, Bijenička cesta 54, HR-10000 Zagreb, Croatia; dzilic@irb.hr (D.Ž.); Ana.Santic@irb.hr (A.Š.); Juraj.Ovcar@irb.hr (J.O.); Ivor.Loncaric@irb.hr (I.L); Zoran.Stefanic@irb.hr (Z.Š); Alen.Bjelopetrovic@irb.hr (A.B); jpopovic@irb.hr (J.P.)

* Correspondence: ipicek@mef.hr (I.P.); jpopovic@irb.hr (J.P.)

Contents:

Figure S1. Structural formula of *N*-benzylpyridinium-4-oxime cation ($BPA4^+$).

Figure S2: Crystal packing of $(BPA4)_4[Fe(CN)_6]$.

Table S1: Crystal data and structure refinement for $(BPA4)_4[Fe(CN)_6]$.

Figure S3: a) 2D Fingerprints plots and Hirshfeld surfaces of hexacyanoferrate ion mapped for b) d_{norm}, and c) curvature of hexacyanoferrate(II) ion in crystal structures of up: $(BPA4)_4[Fe(CN)_6]\cdot 10H_2O$ and down: $(BPA4)_4[Fe(CN)_6]$.

Interpretation of Hirshfeld surfaces shown in Fig. S3.

Figure S4: Isosurface of value 0.3 of the ground state electronic density for a) (BPA4)4[Fe(CN)6]·10H2O and b) (BPA4)4[Fe(CN)6]. In (BPA4)4[Fe(CN)6]·10H2O, no charge-transfer is observed between the CN- ligands and the water molecules (red dashed circle), while hydrogen bonds form between all ligands and the BPA4 cations (green dashed circles). For visual clarity, only a portion of the isosurfaces and the atoms in the unit cell is shown.

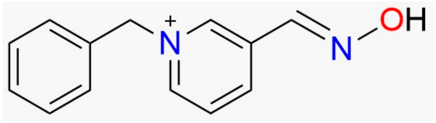

Figure S1. Structural formula of *N*-benzylpyridinium-4-oxime cation ($BPA4^+$).

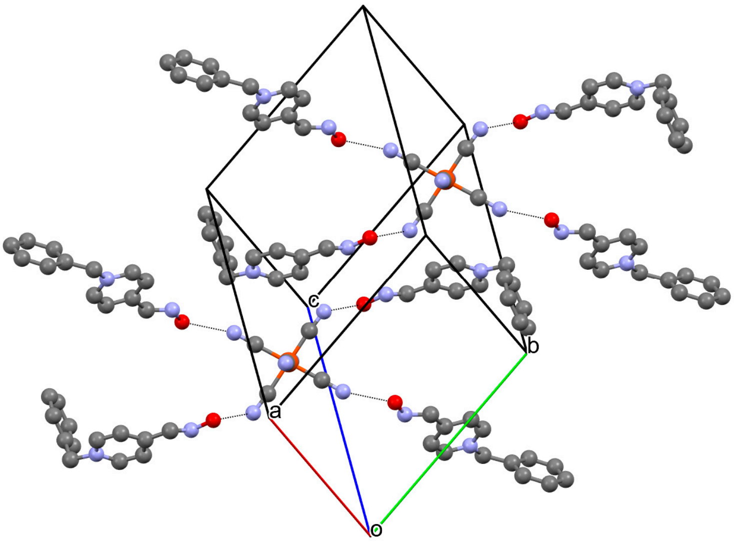

Figure S2. Crystal packing of $(BPA4)_4[Fe(CN)_6]$.

Table S1. Crystal data and structure refinement for $(BPA4)_4[Fe(CN)_6]$.

Empirical formula	$C_{29}H_{26}N_7O_2Fe_{0.5}$
Formula weight	532.49
Temperature/K	293(2)
Crystal system	triclinic
Space group	*P* -1
a/Å	10.5095(18)
b/Å	12.143(3)
c/Å	12.576(4)
α/°	63.16(3)
β/°	67.46(2)
γ/°	76.523(17)
Volume/Å^3	1319.1(5)
Z	2
ρ_{calc}g/cm^3	1.341
μ/mm^{-1}	2.796
F(000)	556.0
Crystal size/mm^3	0.12 × 0.1 × 0.05
Radiation	Cu Kα (λ = 1.54184)
2Θ range for data collection/°	8.182 to 103.63
Index ranges	$-10 \le h \le 10$, $-11 \le k \le 12$, $-12 \le l \le 12$
Reflections collected	5378
Independent reflections	2871 [R_{int} = 0.0933, R_{sigma} = 0.1684]
Data/restraints/parameters	2871/0/351
Goodness-of-fit on F^2	1.109
Final R indexes [I>=2σ (I)]	R_1 = 0.1090, wR_2 = 0.1581
Final R indexes [all data]	R_1 = 0.1911, wR_2 = 0.1887
Largest diff. peak/hole / e Å^{-3}	0.42/-0.20

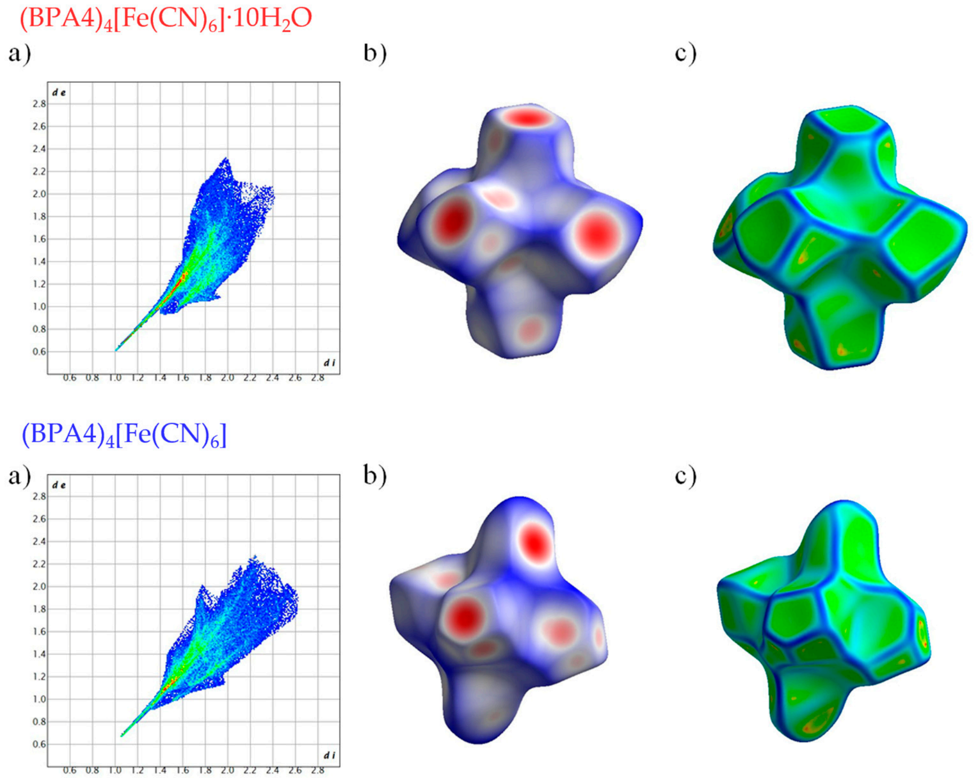

Figure S3. a) 2D Fingerprints plots and Hirshfeld surfaces of hexacyanoferrate ion mapped for b) d_{norm}, and c) curvature of hexacyanoferrate(II) ion in crystal structures of up: $(BPA4)_4[Fe(CN)_6]\cdot 10H_2O$ and down: $(BPA4)_4[Fe(CN)_6]$.

As shown in Figure S3, d_{norm} mapping indicates strong OH···N hydrogen bonding interactions as primary interactions between hexacyanoferrate anion and surrounding molecules in both structures seen as bright red spots. The analysis of the 2D fingerprint plots from Hirshfeld surface analysis revealed a higher ratio of OH···N interactions (77.2%) in the $(BPA4)_4[Fe(CN)_6]$ than in $(BPA4)_4[Fe(CN)_6]\cdot 10H_2O$ (72.0%). The dominant interaction in both of the structures is R–OH···N interaction indicated by a significant spike. In the case of $(BPA4)_4[Fe(CN)_6]\cdot 10H_2O$, there are two types of OH···N interactions: R–OH···N (~54%), which corresponds to the top, narrow part of the spike, and H–OH···N (~18%) which corresponds to an upper, slightly broader part of the peak.

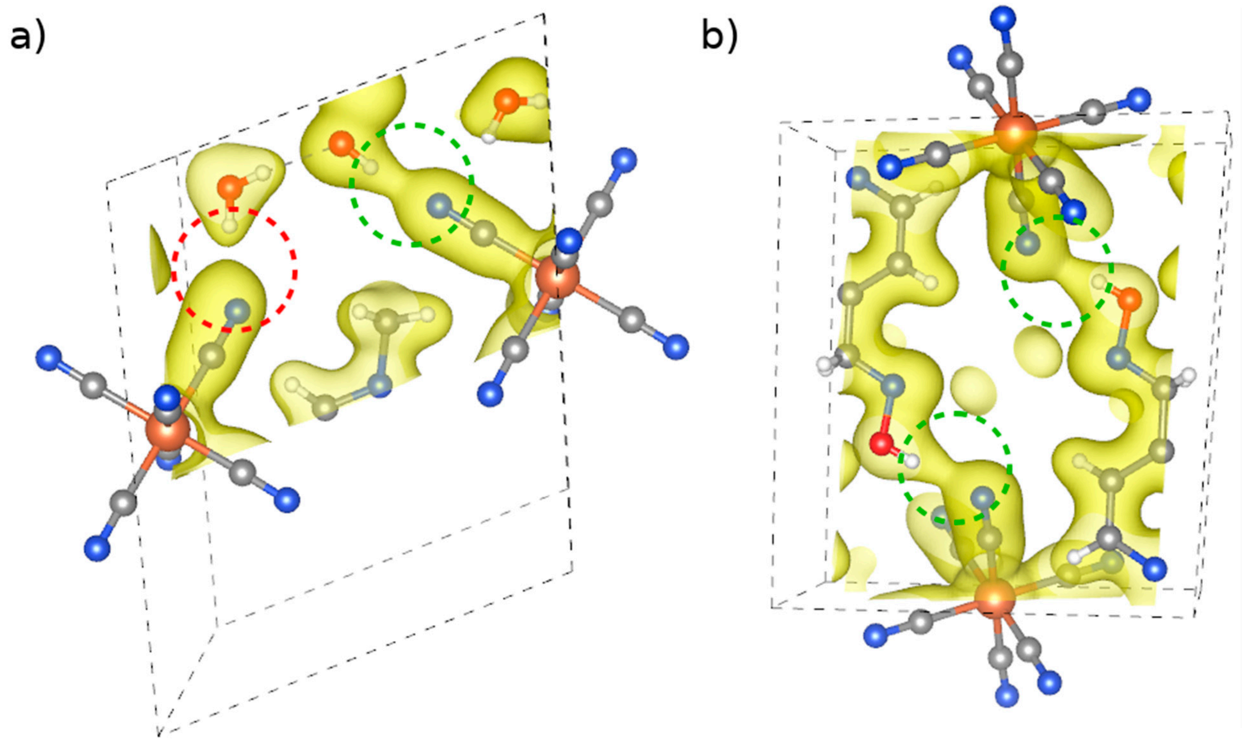

Figure S4: Isosurface of value 0.3 of the ground state electronic density for a) $(BPA4)_4[Fe(CN)_6]\cdot10H_2O$ and b) $(BPA4)_4[Fe(CN)_6]$. In $(BPA4)_4[Fe(CN)_6]\cdot10H_2O$, no charge-transfer is observed between the CN^- ligands and the water molecules (red dashed circle), while hydrogen bonds form between all ligands and the BPA4 cations (green dashed circles). For visual clarity, only a portion of the isosurfaces and the atoms in the unit cell is shown.
